# Elevated Expression and Pro-Inflammatory Activity of IL-36 in Patients with Systemic Lupus Erythematosus

**DOI:** 10.3390/molecules201019588

**Published:** 2015-10-27

**Authors:** Man Chu, Chun Kwok Wong, Zhe Cai, Jie Dong, Delong Jiao, Ngar Woon Kam, Christopher Wai Kei Lam, Lai Shan Tam

**Affiliations:** 1 Department of Chemical Pathology, The Chinese University of Hong Kong, Prince of Wales Hospital, Shatin, NT, Hong Kong, China; E-Mails: shaanxichuman@gmail.com (M.C.); ck-wong@cuhk.edu.hk (C.K.W.); caifranklin@163.com (Z.C.); dongjie0805@163.com (J.D.); j.dl@foxmail.com (D.J.); 2 Shenzhen Research Institute, The Chinese University of Hong Kong, Shenzhen 518057, China; E-Mail: yvonne_kam@yahoo.com.hk; 3 State Key Laboratory of Phytochemistry and Plant Resources in West China, Institute of Chinese Medicine, The Chinese University of Hong Kong, Hong Kong, China; 4 Department of Medicine and Therapeutics, The Chinese University of Hong Kong, Prince of Wales Hospital, Shatin, Hong Kong, China; 5 State Key Laboratory of Quality Research in Chinese Medicine, Macau Institute for Applied Research in Medicine and Health, Macau University of Science and Technology, Taipa, Macau; E-Mail: wklam@must.edu.mo

**Keywords:** cytokines, IL-36, regulatory B lymphocytes, systemic lupus erythematosus

## Abstract

We investigated the expression and proinflammatory activity of interleukin (IL)-36 in patients with systemic lupus erythematosus (SLE). The expression level of IL-36, its putative receptors and the frequency of CD19^+^CD24^high^CD27^+^ regulatory B (Breg) lymphocytes of peripheral blood from 43 SLE patients and 16 normal control (NC) subjects were studied using ELISA and flow cytometry. Plasma cytokines/chemokines and *ex vivo* productions of cytokine/chemokine from peripheral blood mononuclear cells (PBMC) stimulated with recombinant IL-36 were determined by Luminex multiplex assay. Plasma concentrations of IL-36α, IL-36γ and the proportions of circulating IL-36R-positive CD19^+^ B lymphocytes in total B lymphocytes and PBMC were significantly increased in active SLE patients compared with NC (all *p* < 0.05). Plasma IL-36α and IL-36γ correlated positively with SLE disease activity and elevated plasma IL-10 concentration (all *p* < 0.05). The frequencies of circulating Breg lymphocytes in total B lymphocytes and PBMC were significantly decreased in both inactive and active SLE patients compared with NC (all *p* < 0.01). The frequency of Breg lymphocytes in total B lymphocytes correlated negatively with the proportion of IL-36R-positive B lymphocytes (*p* < 0.05). IL-36α exerted substantial proinflammatory effect in PBMC from SLE patients by inducing the production of IL-6 and CXCL8. Upon stimulation with IL-36α and IL-36γ, *ex vivo* productions of IL-6 and CXCL8 were significantly increased in SLE patients compared with NC (all *p* < 0.05). This cross-sectional study demonstrated that over expression of circulating IL-36α may exert a proinflammatory effect as observed in human SLE.

## 1. Introduction

Systemic lupus erythematosus (SLE) is a chronic autoimmune disease characterized by the dysregulated activation of both B and T lymphocytes and the subsequent overproduction of auto- antibodies and proinflammatory cytokines [1]. It is generally believed that SLE is associated with abnormal cytokine levels, including increased T helper cell type 1 (Th1) cytokine [interferon (IFN)-γ], Th2 cytokine [interleukin (IL)-4], which stimulate antibodies production, and Th17 cytokines (e.g., IL-17) as well as inflammatory IL-1β, IL-6 and IL-18 that may contribute to the pathogenesis of SLE [2,3,4,5,6]. IL-10, primarily produced by monocytes, Th2, regulatory T and B lymphocytes, is elevated in SLE patients and correlated well with SLE disease activity [7]. These abnormalities indicated a rather complex cytokines network which required further investigations in human SLE.

IL-36 is a newly named cytokine of the IL-1 cytokine family comprising three members, IL-36α, IL-36β and IL-36γ (previously designated as IL-1F6, IL-1F8 and IL-1F9, respectively) [8,9]. All three IL-36 isoforms bind to a heterodimer consisting of IL-36 receptor (IL-36R, previously called IL-1Rrp2/IL-1R6) and IL-1RAcP, a common co-receptor, which together activate intracellular signals similar to those induced by IL-1. IL-36R antagonist (IL-36Ra) binds to IL-36R without inducing any signaling response, thereby acting as a natural inhibitor similar to IL-1Ra/IL-1 system [10]. At present, it is believed that IL-36 expressed in a few human tissues in a restricted manner, primary by keratinocytes in the skin and other epithelial cell types upon exposure to pathogens [11]. Evidences based on the expression pattern of IL-36 and its receptor in human psoriasis and mouse models have defined a crucial pathological role for IL-36 in skin inflammation, partly by acting on the crosstalk between keratinocytes and inflammatory dendritic cells (DC) [12,13]. The relationship between Th17 cytokines and IL-36 has also been previously established in human keratinocyte culture [14]. IL-36 expression can be up-regulated by Th17 cytokines and in turn, IL-36 production can regulate the expression and enhance the function of Th17 cytokines in an autocrine manner [14]. Notably, Vigne *et al.* found that IL-36 can skew the differentiation of naive mouse T cells into IFN-γ-producing Th1 cells [15]. Moreover, they demonstrated that IL-36 can promote the maturation of DC and stimulate mouse bone-marrow-derived DC to produce inflammatory cytokines at a higher level than other IL-1 family members, highlighting its potential role in bridging the innate and adaptive immunity [16]. 

Regulatory B (Breg) lymphocytes have an important role in suppressing auto-reactive and pathogen-driven immune response by secreting anti-inflammatory cytokine IL-10 [17]. Dysregulation of Breg lymphocytes may be involved in the development of various autoimmune diseases including SLE [18]. In humans, different B cell subsets are enriched in Breg cells, mainly including CD24^high^CD27^+^ and CD24^high^CD38^high^ B cell subsets. Previous studies have reported the lack of suppressive capacity regarding CD19^+^CD24^high^CD38^high^ Breg lymphocytes in SLE patients and CD19^+^CD24^high^CD27^+^ Breg lymphocytes in Graves’s disease and graft-versus-host disease [19,20,21]. However, the role of CD19^+^CD24^high^CD27^+^ Breg lymphocytes and the novel proinflammatory cytokine IL-36 in the regulation of human SLE remains unknown. In this context, we investigated the expression pattern and function of IL-36 and IL-36R in peripheral blood of SLE patients, in an attempt to elucidate the immunological roles of IL-36 and CD19^+^CD24^high^CD27^+^ Breg lymphocytes and their contribution in the cytokine network of SLE.

## 2. Results and Discussion

### 2.1. Demographical and Clinical Characteristics of SLE Patients and NC

Forty-three Chinese SLE patients were recruited and divided into two groups according to disease activity. Sixteen age- and sex-matched healthy Chinese volunteers were recruited as controls. Demographics and clinical characteristics are summarized in Table 1. Plasma albumin concentration was significant lower in inactive and active SLE patients (38 ± 7 g/L, and 31 ± 5 g/L, respectively) compared with NC (45 ± 2 g/L, both *p* < 0.01). Similarly, patients with inactive and active SLE had lower plasma total protein concentration compared with NC (both *p* < 0.01). Clinical manifestations at the time of study included nephritis (34/43, 79.1%), serositis (12/43, 27.9%), hematologic derangement (11/43, 25.6%) and arthritis (17/43, 39.5%) of all the studied SLE patients. 

**Table 1:** Demographic and clinical characteristics of SLE patients and NC.

	SLE Patients (*n* = 43)		Normal Control (*n* = 16)
	Inactive SLE (*n* = 22)	Active SLE (*n* = 21)	
**Demographic Characteristics**			
Female sex (n) (%)	20 (90.9)	13 (95.2)	14 (87.5)
Age at study (year)			
Mean(SD)	45.2 (9.5)	48.2 (10.5)	41.4 (12.5)
Range	24–59	23–60	23–57
**Clinical Features**			
SLE duration (year)			
Mean(SD)	14.8 (5.9)	16.6 (7.6)	N/A
Range	1–28	10–26	N/A
SLICC median(IQR)	0 (0–1)	0 (0–3)	N/A
SLEDAI median(IQR)	2 (2–4)	8 (6–16)	N/A
Flare (*n*) (%)	5 (22.7)	16 (76.2)	N/A
**Serological Features**			
Serum complement C3 (g/L) mean (SD)	0.80 (0.24)	0.62 (0.19)	N/A
Serum complement C4 (g/L) mean (SD)	0.15 (0.09)	0.08 (0.05)	N/A
Anti-dsDNA >1000 IU/mL (n) (%)	2 (9.1)	3 (14.2)	N/A
Anti-dsDNA <60 IU/mL (n) (%)	3 (13.6)	1 (4.8)	N/A
Anti-dsDNA titer (IU/mL) mean (SD)	268.4 (185.1)	409.6 (201.7)	N/A
Plasma urea (mmol/L) mean (SD)	5.8 (1.7) *	6.3 (1.5) **	4.7 (1.2)
Plasma creatinine (μmol/L) mean (SD)	70.2 (28.0)	79.5 (30.3) *	61.7 (12.9)
Plasma total protein (g/L) mean (SD)	70.3 (10.9) **	66.1 (8.7) ***	77.6 (3.3)
Plasma albumin (g/L) mean (SD)	38.2 (7.2) **	31.9 (5.2) ***	45.6 (2.3)
**Major Organ System Involvement (*n*) (%)**			
0	5 (22.7)	0 (0)	N/A
1	10 (45.4)	7 (33.3)	N/A
≥2	7 (32.9)	14 (66.7)	N/A
**Clinical Manifestation (*n*) (%)**			
Nephritis	13 (59.1)	21 (100.0)	N/A
Serositis	5 (22.7)	7 (33.3)	N/A
Hematologic	5 (22.7)	6 (28.6)	N/A
Arthritis	8 (38.1)	9 (42.8)	N/A
**Current Immunosuppressive Therapy**			
Treatment with prednisolone			
Patients (n) (%)	17 (77.3)	19 (90.5)	N/A
Daily dose (mg) mean (SD)	7.5 (3.5)	9.2 (5.7)	N/A
Treatment with hydroxychloroquine			
Patients (n) (%)	9 (40.9)	10 (47.6)	N/A
Daily dose (mg) mean (SD)	236.7 (95.1)	300.0 (115.5)	N/A
Treatment with mycophenolatemofetil			
Patients (n) (%)	10 (45.4)	10 (47.6)	N/A
Daily dose (mg) mean (SD)	883.3 (458.1)	1000.0 (250.0)	N/A
Treatment with lisinopril			
Patients (n) (%)	11 (50.0)	13 (61.9)	N/A
Daily dose (mg) mean (SD)	9.6 (7.4)	14.4 (5.3)	N/A
Treatment with azathioprine			
Patients (n) (%)	4 (18.2.7)	7 (33.3)	N/A
Daily dose (mg) mean(SD)	58.3 (38.2)	100.0 (50.0)	N/A

SD, standard deviation; N/A, not applicable; Inactive SLE: SLEDAI < 6, Active SLE: SLEDAI ≥ 6; SLEDAI, systemic lupus erythematosus disease activity index; SLICC, Systemic Lupus International Collaborating Clinics Score; Major organ system involvement includes: musculoskeletal, kidney, skin, heart and hematologic (hemolytic anemia, platelet < 100,000/μL); ‘‘Flare’’ is defined as increase in the SLEDAI score by 3 or more. * *p* < 0.05, ** *p* < 0.01 and *** *p* < 0.001 when compared with normal control.

### 2.2. Elevated Plasma IL-36 Correlated Positively with SLE Disease Activity and Plasma IL-10

**Figure 1:**
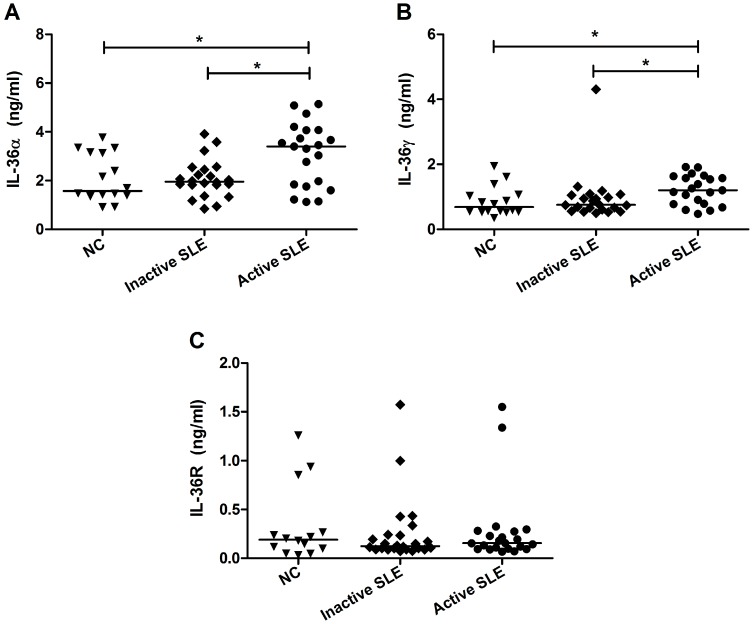
Comparison of plasma IL-36 concentrations between SLE patients and NC. Plasma concentrations of (**A**) IL-36α; (**B**) IL-36γ and (**C**) IL-36R from inactive (*n* = 22) and active (*n* = 21) SLE patients and NC (*n* = 16) were measured using ELISA. Results are presented as scatter plots with median. Statistical significances were indicated by * *p* < 0.05 (Mann-Whitney U test).

**Figure 2:**
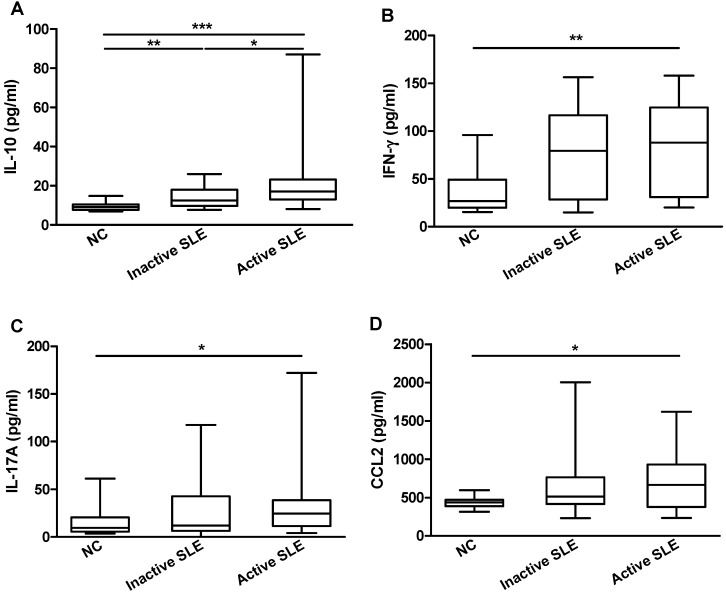
Comparison of plasma cytokines/chemokines concentrations between SLE patients and NC. Plasma concentrations of (**A**) IL-10; (**B**) IFN-γ; (**C**) IL-17A and (**D**) CCL2 from inactive (*n* = 22) and active (*n* = 21) SLE patients and NC (*n* = 16) were measured using Milliplex MAP assay kit. Results are presented as box and whisker plots with median (interquartile range). Statistical significances were indicated by * *p* < 0.05, ** *p* < 0.01 and *** *p* < 0.001 (Mann-Whitney U test).

Given the proinflammatory nature of IL-36 in psoriasis, the identification of these new members raised intriguing possibilities that IL-36 might also be involved in SLE. We examined the plasma concentrations of these novel cytokines and their receptor by ELISA in inactive (*n* = 22), active (*n* = 21) SLE patients and NC subjects (*n* = 16). There were detectable IL-36α, IL-36γ and IL-36R present in the plasma of both NC and SLE patients. As shown in Figure 1, a comparable plasma IL-36R concentration was displayed by all SLE patients, whereas the levels of IL-36α and IL-36γ were significantly higher in active SLE patients than those in NC (3.6 ± 0.2 *vs.* 2.0 ± 0.2 ng/mL and 1.2 ± 0.1 *vs.* 0.7 ± 0.1 ng/mL, respectively, both *p* < 0.05). We also found higher concentrations of plasma IL-10, IFN-γ, IL-17A and CCL2 in SLE patients compared with NC (all *p* < 0.05, Figure 2).

Since SLE is associated with low serum concentrations of complements C3 and C4, we further investigated the correlations of plasma IL-36 with SLEDAI score, serum complements C3 and C4 as well as several common cytokines/chemokines in all the studied SLE patients (*n* = 43). As illustrated in Table 2, IL-36α and IL-36γ correlated positively with SLEDAI score (r = 0.382 and r = 0.327, respectively, both *p* < 0.05). IL-36γ, but not IL-36α, correlated negatively with serum C4 (r = −0.339, *p* < 0.05). We further found that plasma IL-36α and IL-36γ concentrations correlated positively with the elevated IL-10 concentrations (r = 0.306 and r = 0.338, respectively, both *p* < 0.05). No obvious correlations of IL-36 with other common cytokines/chemokines (IFN-γ, IL-17A and CCL2) were found in all the SLE patients (*p* > 0.05, data not shown).

**Table 2:** Correlations of plasma IL-36 concentrations with SLE disease activity and IL-10.

	IL-36α (ng/mL)		IL-36γ (ng/mL)	
	r Value	*p* Value	r Value	*p* Value
SLEDAI	0.382	0.011 *	0.327	0.025 *
C3 (g/L)	−0.147	0.342	−0.211	0.154
C4 (g/L)	−0.138	0.371	−0.339	0.019 *
IL-10 (pg/mL)	0.306	0.046 *	0.338	0.023 *

N = 43; Non-parametric Spearman’s test was used to assess the correlations. r = Spearman’s correlation coefficient. * *p* < 0.05

### 2.3. Elevated Proportion of CD19^+^ B Cells Expressed with IL-36R in SLE Patients

**Figure 3:**
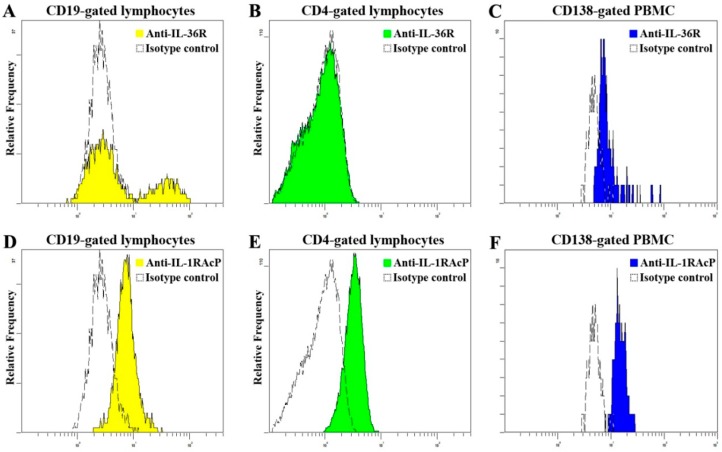

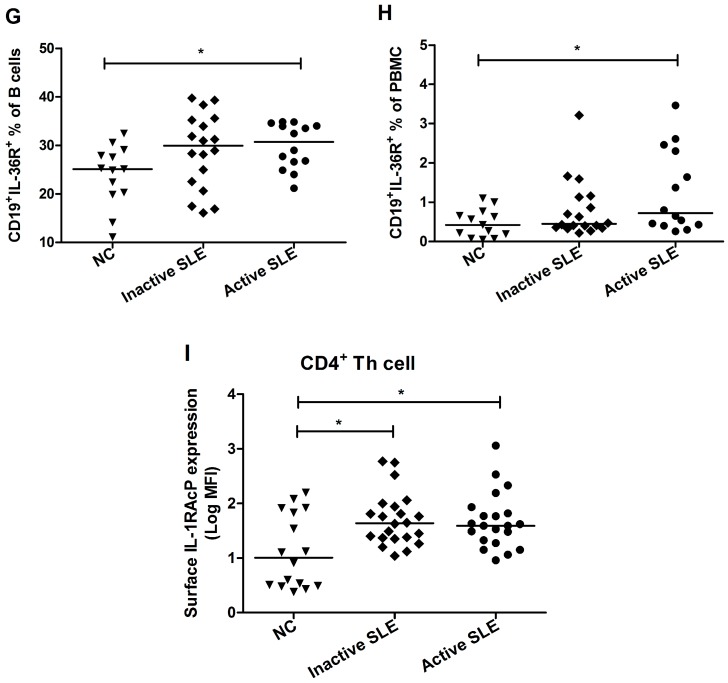
Expression profile of IL-36 receptors on the surface of immune cells. (**A**–**C**) Representative histograms of IL-36R expression on CD19^+^ B cells, CD4^+^ Th cells and CD138^+^ plasma cells in SLE patients and NC; (**D**–**F**) Representative histograms of IL-1RAcp expression on CD19^+^ B cells, CD4^+^ Th cells and CD138^+^ plasma cells in SLE patients and NC; (**G**–**I**) The proportion of IL-36R^+^ B cells in total B cells and PBMC from inactive (*n* = 18), active (*n* = 14) SLE patients and NC (*n* = 13), and the expression level of IL-1RAcP on CD4^+^ Th cells from inactive (*n* = 22), active (*n* = 21) SLE patients and NC (*n* = 16) were detected using flow cytometry. Results are presented as scatter plots with median of the proportion or mean fluorescence intensity (MFI) subtracting corresponding isotypic controls. Statistical significance indicated by * *p* < 0.05 when compared with NC (Mann-Whitney U test).

IL-36 cytokines mediate signal through IL-36R and the recruitment of the co-receptor IL-1RAcP [10]. Flow cytometric analysis showed that IL-36R was strongly expressed on the surface of CD138^+^ plasma cells (Figure 3C) and certain population of CD19^+^ B lymphocytes (Figure 3A), while its expression was absent on the cell surface of CD4^+^ Th lymphocytes (Figure 3B). Alternatively, IL-1RAcp was expressed on the surface of CD19^+^ B lymphocytes (Figure 3D), CD4^+^ Th lymphocytes (Figure 3E) and CD138^+^ plasma cells (Figure 3F). Firstly, we examined the proportion IL-36R^+^ B cells within CD19^+^ B cells gating. The representative dot plots of IL-36R^+^ B lymphocytes are shown in Supplementary Figure S1. By comparing with NC (*n* = 13), higher IL-36R^+^ B cells % in total B cells (29.9% ± 1.3% *vs* 23.9% ± 1.7%, *p* < 0.05) was observed in active SLE patients (*n* = 14), but not inactive SLE patients (*n* = 18, Figure 3G). Similarly, higher IL-36R^+^ B cells % among the total number of PBMC were presented comparing with NC (1.3% ± 0.3% *vs.* 0.5% ± 0.1%, *p* < 0.05, Figure 3H). As shown in Figure 3I, the expression level of IL-1RAcP on the surface of CD4^+^ Th lymphocytes was significantly upregulated in both active (*n* = 21) and inactive (*n* = 22) SLE patients (1.7% ± 0.1% and 1.7% ± 0.1% *vs.* 1.1% ± 0.2%, respectively, both *p* < 0.05) compared with NC (*n*=16).

There was no significant difference of IL-1RAcp expression level on CD19^+^ B cells and CD138^+^ plasma cells of SLE patients comparing with controls, and similar expression level of IL-36R on CD138^+^ plasma cells was found between SLE patients and NC (all *p* > 0.05, Supplementary Figure S2).

### 2.4. Decrement of CD19^+^CD24^high^CD27^+^ Breg Lymphocytes in SLE Patients 

**Figure 4:**
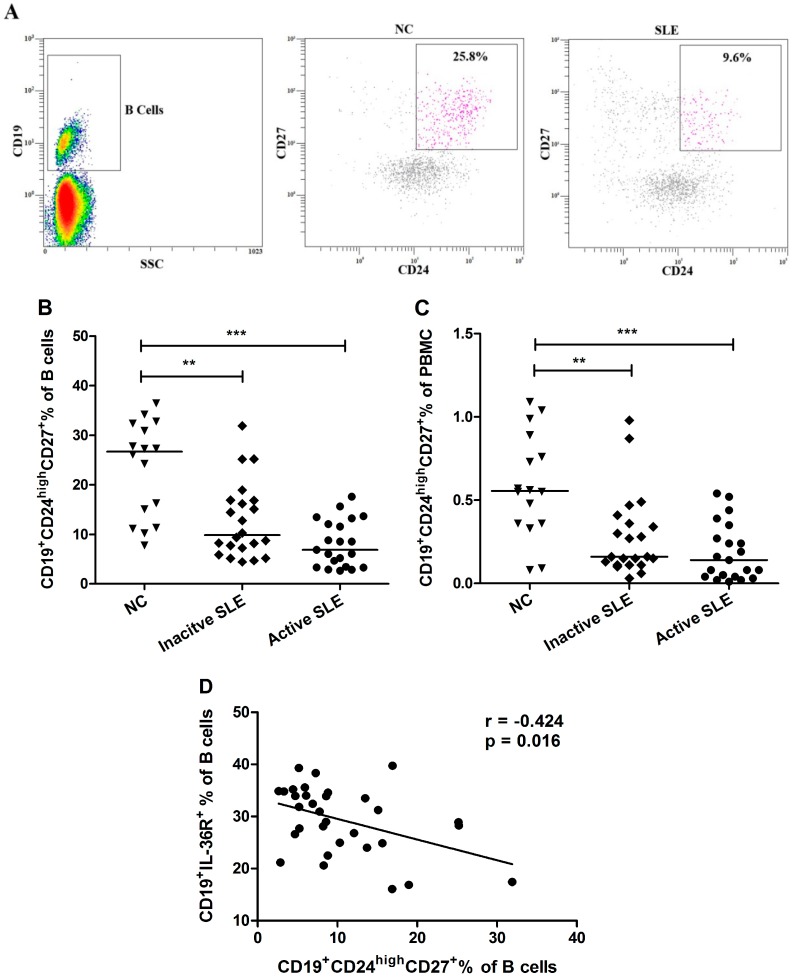
Comparison of circulating CD19^+^CD24^high^CD27^+^ Breg lymphocytes frequency between SLE patients and NC. (**A**) Representative dot plots are shown for the CD19^+^CD24^high^CD27^+^ Breg lymphocytes gated from CD19^+^ B cells in SLE patients and NC; (**B**,**C**) The proportion of circulating Breg lymphocytes from inactive (*n* = 22) and active (*n* = 21) SLE patients and NC (*n* = 16) in total B cells and in PBMC were determined by flow cytometry. Results are presented as scatter plots with median. (**D**) Correlation between % CD19^+^IL-36R^+^ B cells and % CD19^+^CD24^high^CD27^+^ Breg cells was analyzed with Spearman’s test. Statistical significances are indicated by ** *p* < 0.01 and *** *p* < 0.001 when compared with NC (Mann-Whitney U test).

Human circulating Breg lymphocytes are defined as CD19^+^ B cells with surface expression of CD24 and CD27. Given the potential contribution of Breg in autoimmunity [18], we evaluated the frequency of Breg cells in SLE patients. In NC (*n* = 16), Breg lymphocytes composed 23.8% ± 3.3% of B lymphocytes and 0.6% ± 0.1% of PBMC, while in inactive (*n* = 22) and active (*n* = 21) SLE patients, corresponding proportions were 13.1% ± 2.1% and 9.5% ± 1.7% of B lymphocytes, respectively, and 0.3% ± 0.1% and 0.2% ± 0.1% of PBMC, respectively. The frequencies of circulating CD19^+^CD24^high^CD27^+^ Breg lymphocytes of total B cells and of PBMC were significantly decreased in active and inactive SLE patients compared with NC (Figure 4A–C, all *p* < 0.01).

Furthermore, we were interested in studying the correlations among the frequency of circulating Breg lymphocytes and the proportion of IL-36R^+^ B cells in all the studied patients. As shown in Figure 4D, the frequency of CD19^+^CD24^high^CD27^+^ Breg cells in total B cells correlated negatively with the proportion of IL-36R^+^CD19^+^ B cells (r = −0.424, *p* = 0.016; *n* = 32) in SLE patients.

### 2.5. IL-36 Promotes the Production of Cytokine/Chemokine in PBMC

The above findings prompted us to investigate the ability of IL-36 to induce proinflammatory and/or anti-inflammatory cytokines and chemokines involved in immune and inflammatory responses. We did not observe any significant difference of *ex vivo* production of cytokine/chemokine between inactive and active SLE patient groups, however, PBMC from both SLE patients and NC could be activated by human recombinant IL-36α or IL-36γ (both at 1 μg/mL, Table 3). In SLE patients, compared with unstimulated control, IL-36α induced markedly higher production of inflammatory IL-6 and CXCL8 (*p* < 0.05 and *p* < 0.01, respectively). In addition, compared with NC, PBMC from SLE patients constitutively produced higher level of IL-6 and CXCL8 (both *p* < 0.01). Upon stimulation with IL-36α and IL-36γ, IL-6 and CXCL8 showed significantly higher concentrations in the culture supernatant of PBMC from SLE patients (all *p* < 0.05). Notably, IL-36α and IL-36γ failed to upregulate the production of Th1 cytokine IFN-γ in the PBMC culture of both NC and SLE patients (data not shown). Moreover, the production of IL-17 was barely detectable in all the treated or untreated control groups.

**Table 3:** *Ex vivo* induction of cytokine/chemokine from PBMC stimulated with IL-36.

Cytokine/Chemokine	Group	Basal Median (IQR) (pg/mL)	Post stimulation with IL-36α Median (IQR) (pg/mL)	Post stimulation with IL-36γ Median (IQR) (pg/mL)
IL-6	NC	2.3 (0–3.9)	5.6 (1.9–10.4) ^#^	3.6 (1.5–6.6)
	SLE	26.5 (3.6–43.4) ***	44.4 (9.9–90.96) ***^,^^&^	35.1 (7.3–65.4) ***
CXCL8	NC	548.3 (399.1–676.1)	1347.0 (785.6–2586.0) ^###^	808.6 (505.2–1300.0) ^#^
	SLE	1288.0 (1145.0–4487.0) **	2661.0 (1145.0–4487.0) *^,&&^	1510.0 (678.1–3673) *
IFN-γ	NC	11.9 (10.4–19.4)	16.1 (11.9–27.2)	14.3 (6.6–23.6)
	SLE	14.5 (9.3–20.2)	17.4 (11.3–21.6)	17.4 (11.9–23.0)
IL-17A	NC	UD	UD	UD
	SLE	UD	UD	UD

The culture supernatant was derived from PBMC cultured with medium in the presence of human recombinant IL-36α and IL-36γ (1 μg/mL) for 24 h. * *p* < 0.05, ** *p* < 0.01 and *** *p* < 0.001 when compared with NC; ^#^ *p* < 0.05 and ^###^ *p* < 0.001 when compared with untreated control in NC group; ^&^ *p* < 0.05 and ^&&^ *p* < 0.01 when compared with untreated control in SLE group. UD: undetectable.

Our present study has examined the detailed expression pattern of IL-36 and its receptor, as well as the functional consequences of IL-36R signaling way in SLE patients. We have demonstrated higher plasma concentrations of IL-36α and IL-36γ in active SLE patients compared with NC (Figure 1). Furthermore, the elevated IL-36α and IL-36γ correlated positively with SLEDAI and the elevated plasma IL-10, while IL-36γ correlated negatively with serum complement C4 (Table 2), suggesting that plasma IL-36 values may reflect SLE disease activity. After statistical analysis, we observed that there were no significant effects of drug treatment on IL-36 expression in SLE patients (all *p* > 0.05). Prompted by the high expression of plasma IL-36α and IL-36γ in SLE patients, we investigated the activity of IL-36R on immune cells in peripheral blood of SLE patients. It has been reported that mouse CD4^+^, but not CD8^+^ T cells, respond to IL-36 in an IL-36R-dependent manner and IL-36 synergizing with IL-12 can drive a potent murine Th1 differentiation [15]. However, in contrast to mouse, we found that human CD4^+^ Th cells do not express IL-36R (Figure 3B), which is consistent with a previous study [22]. Importantly, about 30% B cells displayed surface expression of IL-36R in peripheral blood of SLE patients. The proportion of IL-36R^+^ B cells of total B cells and PBMC showed a small but significant elevation in SLE patients compared with NC (Figure 3G–H). Collectively, the increased levels of IL-36 and IL-36R may imply a potential role of IL-36/IL-36R signaling system in the inflammatory pathogenesis of chronic autoimmune disease SLE.

IL-36/IL-36R signaling complex has been previously proved to exert proinflammatory effects contributing to the pathogenesis of skin inflammation [23,24]. In addition, overexpressed of circulating and tissue levels of IL-36α were also reported in autoimmune disease primary Sjögren’s syndrome [25]. Some studies have shown that IL-36 does not participate in the inflammatory progression of experimental arthritis though up-regulated IL-36α was found in the synovial tissue of patients with rheumatoid arthritis [26,27,28]. Our present result of non-significant difference of plasma IL-36 concentrations between inactive SLE patients (SLEDAI < 6) and normal controls (Figure 1A) was actually in concordance with previous publication of Zhang *et al.* [29] using SLE patients with SLEDAI < 6. Nevertheless, we have demonstrated a significant higher plasma IL-36α and IL-36γ concentrations in active SLE patients (SLEDAI ≥ 6) compared with NC (Figure 1A,B). Moreover, in all the studied SLE patients, these cytokines were found to be correlated positively with disease severity and plasma IL-10, which is considered to be a biomarker in human SLE [7], providing evidence that IL-36 might be involved in the pathogenesis of human SLE, especially during the late-stage of the disease. Although skin is often affected by SLE, there was no significant difference of plasma IL-36 concentrations among SLE patients with or without malar rash and normal controls in this study (all *p* > 0.05).

Emerging evidence has suggested that Breg subset could down-modulate adaptive or innate immune responses in mice and humans. Our present study, for the first time, characterized the CD19^+^CD24^high^CD27^+^ Breg subset in patients with SLE. We found that the frequencies of Breg lymphocytes were significantly decreased in both inactive and active SLE patients compared with NC (Figure 4). Furthermore, correlation study revealed that the frequency of CD19^+^CD24^high^CD27^+^ Breg lymphocytes correlated negatively with the proportion of IL-36R^+^ B cells (Figure 4D). Although we did not find a significant correlation of plasma IL-36 concentrations with Breg cell subpopulation (*p* > 0.05), the decreased CD19^+^CD24^high^CD27^+^ Breg lymphocytes in SLE patients imply its important contribution in regulating immune response and there may be a reverse inter-regulation between circulating inflammatory IL-36 and anti-inflammatory Breg subpopulations. We found IL-36R expressed on certain proportion of B cells (Figure 3A and Supplementary Figure S1), but we could not identify which B cell subset expressing IL-36R using B cell markers (IgD, CD38, CD24 and CD27) with PBMC purified from human buffy coat of normal blood donor (data not shown). However, the evaluation of IL-36R^+^ B cell subset such as Breg and how IL-36 modulates IL-10-producing-Breg lymphocytes in SLE patients is required for further investigation. 

Our functional experiments revealed that IL-36α and IL-36γ, both of which are known to exhibit the same biological activities [11], have functional consequences in SLE patients. IL-36α and IL-36γ can stimulate PBMC to produce proinflammatory IL-6 and CXCL8 in NC and/or SLE patients (Table 3). Such direct stimulatory effects of IL-36 have also been reported on human keratinocyte, mouse splenocytes, bone marrow-derived DC and CD4^+^ Th cells [16,30]. Furthermore, the *ex vivo* productions of IL-6 and CXCL8 from PBMC of SLE patients were markedly higher which may lead to a more complex cytokine inter-regulation network for the pathogenesis of human SLE. It further supported the correlation between IL-36 and SLEDAI. Previous study showed that IL-36 could skew differentiation of naive mouse T cells toward IFN-γ-producing Th1 cells [15]. In contrary, we observed no enhanced expression of Th1-producing cytokine, IFN-γ, in PBMC culture supernatant upon the exposure to IL-36α and IL-36γ. Moreover, plasma IL-36 seemed not to be related with plasma IFN-γ. This discrepancy could possibly due to the absence of IL-36R on human CD4^+^ Th cells, as demonstrated in Figure 3B and another study [22]. Thus, IL-36 may not participate in Th1 subset differentiation in human. Although we did not observe a direct effect of IL-36 on IL-17 production and a relationship between plasma IL-17 and IL-36, we demonstrated a significant increase of circulating CD3^+^IL-22^+^IL-17^+^ T lymphocytes in SLE patients in the present study (Supplementary Figure S3). Further investigation to clarify how IL-36 modulates Th17 cells responses in SLE patients using larger patients sample size would be of interest.

Despite the effect of the use of high recombinant IL-36 concentrations is still unknown, we found that PBMC cultured under optimized conditions after concentration gradient experiment (Supplementary Figure S4) to have intact cell viability for the entire culture period. Interestingly, Magne *et al.* reported that endogenously produced IL-36β is active at much lower doses than recombinant IL-36β [31]. This prompted us to consider the possibility that human IL-36 cytokines may exert its full biological activities via posttranslational modification under physiological conditions [32]. Therefore, such phenomenon might be absent or inefficient in the current *ex vivo* culture by the sole exogenous use of the commercial recombinant human IL-36. 

## 3. Experimental Section

### 3.1. Ethics Statement

Ethics approval for this study was obtained from Clinical Research Ethics Committee of The Chinese University of Hong Kong-New Territories East Cluster Hospitals (reference number: CRE2013.451). All participants provided written and informed consent in accordance with the 1964 Declaration of Helsinki and its later amendments.

### 3.2. SLE Patients and Normal Control (NC) Subjects

Forty-three Chinese patients with SLE were recruited in the rheumatology clinic of the Prince of Wales Hospital for this cross-sectional study. All patients fulfilled the revised American College of Rheumatology (ACR) criteria for SLE [33]. Patients were excluded from the study if they had prior treatment with therapeutic monoclonal antibody or other biologic agents. Patients were divided into two groups according to their disease activity as reflected by SLE disease activity index (SLEDAI). Group 1: patients with inactive disease activity (SLEDAI < 6, *n* = 22) and Group 2: patients with active disease activity (6 ≤ SLEDAI < 24, *n* = 21). Sixteen age- and sex- matched Chinese normal control subjects were also recruited (NC, *n* = 16).

### 3.3. Clinical and Laboratory Parameters 

Patient information on demographic characteristics, clinical features, serological profile and medications were retrieved from medical records. Laboratory investigations including complete blood count, renal and liver function test, and measurement of anti-double stranded DNA (dsDNA) antibody titer, and serum complements C3 and C4 concentrations were performed at study visit. Serum anti-dsDNA titer was measured by ELISA (Euroimmun, Luebeck, Germany). Complements C3 and C4 were assayed using immunonephelometry (Cobas 8000 modular analyzer, Roche Diagnostics Corp., Indianapolis, IN, USA). Major organ system involvement was defined as the involvement of one or more of the following organs including the musculoskeletal, kidney, skin, heart and hematologic system (hemolytic anemia, platelet < 100,000/μL). The prescription of immune- suppressive agents including prednisolone, hydroxychloroquine (HCQ) and mycophenolate mofetil (MMF) was recorded from case notes.

### 3.4. Assays for the Expression Level of Plasma IL-36α, IL-36γ and IL-36R

Plasma concentrations of IL-36α/IL-1F6, IL-36γ/IL-1F9 and IL-36R/IL-1R6 of SLE patients and NC were measured by enzyme-linked immunosorbent assay (ELISA) kits following the manufacturer’s instructions (RayBiotech Inc., Norcross, GA, USA). The intra- and inter-assay coefficient of variability (CV) of each kit is below 10% and 12%, respectively. The sensitivity of IL-36α, IL-36γ and IL-36R ELISA kit is 800, 13 and 24 pg/mL, respectively. Plasma from SLE patients and NC subjects were harvested and stored at −80 °C and all plasma samples were measured on the same ELISA plate. 

### 3.5. Assays for the Expression Level of Plasma Cytokines/Chemokines

Plasma from SLE patients and NC subjects were harvested and stored at −80 °C for subsequent Multiplex Immunoassay of cytokines and chemokines using Luminex multiplex assay kit from Merck Millipore, Corp. (Billerica, MA, USA).

### 3.6. Flow Cytometry 

For the analysis of IL-36 receptors expression, peripheral blood mononuclear cells (PBMC) from SLE patients and NC were purified using Ficoll Plus gradient centrifugation (GE Healthcare Bio-Sciences, Piscataway, NJ, USA) from EDTA venous peripheral blood (20 mL). Indirect immunofluorescent staining was used by incubating PBMC with goat anti-human IL-36R, IL-1RAcp, and normal goat IgG control antibodies (Ab, R & D Systems, Minneapolis, MN, USA) at 4 °C for 30 min in the dark. Then cells were washed and incubated with PE-conjugated donkey anti-goat IgG (R & D Systems), FITC-conjugated anti-human CD4 (clone L200), PerCP-conjugated anti-human CD19 Ab (clone SJ25C1) and APC-conjugated anti-human CD138 Ab (clone MI15, BD Pharmingen Corp., San Diego, CA, USA) at 4 °C for 30 min in the dark. After washing, the expression of cell surface molecules was analyzed by Navios flow cytometry (Beckman Coulter, Brea, CA, USA) and the results were expressed as the percentage or mean fluorescence intensity (MFI). 

To determine the frequency of circulating CD19^+^CD24^high^CD27^+^ Breg lymphocytes, PBMC were purified before surface staining with FITC-conjugated anti-human CD24 (clone ML5), APC-conjugated anti-human CD27 (clone M-T271), PerCP-conjugated anti-human CD19 Ab (clone SJ25C1) and their corresponding isotype control Ab (BD Pharmingen Corp.) at 4 °C for 45 min in the dark. After washing, the proportion of CD19^+^CD24^high^CD27^+^ Breg lymphocytes was quantified by Navios flow cytometer (Beckman Coulter).

### 3.7. Ex vivo Induction of Cytokine/Chemokine from PBMC by Recombinant IL-36

Aliquots of suspended PBMC (1 × 10^5^ cells) in culture medium RPMI1640 supplemented with 10% fetal calf serum (Life Technologies, Grand Island, NY, USA) were dispensed in each well of a 96-well plate (Nalge Nunc International, Naperville, IL, USA). The culture medium used was free of detectable endotoxin (<0.1 EU/mL). The cells were then incubated with or without human recombinant IL-36α and IL-36γ (R & D Systems) at 1 μg /mL for 24 h at 37 °C in a 5% CO_2_ atmosphere. The cell-free supernatant was harvested and stored at −80°C for subsequent Milliplex MAP kit assay reagent (Merck Millipore) with the Bio-Plex 200 suspension array system (Bio-Rad Laboratories, Hercules, CA, USA) of cytokines and chemokines.

### 3.8. Statistical Analysis

Results were expressed as mean ± standard deviation (SD), or median (interquartile range, IQR) if data were not normally distributed. All statistical analysis was performed by the SPSS statistical software for Windows, version 10.1.4 (SPSS, Chicago, IL, USA). For continuous variables, statistical significance was calculated using Mann-Whitney U test. Non-parametric spearman’s test was used to assess the correlation of two variables. All hypotheses were 2-tailed, and *p* values < 0.05 were considered to be significant.

## 4. Conclusions

Taken together, our current cross-sectional patient study demonstrated plasma concentrations of novel proinflammatory cytokine IL-36α, IL-36γ and peripheral IL-36R^+^ B lymphocytes were overexpressed in active SLE patients. Accordingly, we observed that IL-36α exerted substantial proinflammatory effect in SLE patients by inducing the production of IL-6 and CXCL8. Furthermore, the percentage of IL-36R^+^ B lymphocytes correlated negatively with the frequency of Breg lymphocytes, providing evidence that the pathophysiology of SLE may be linked to a complex immune relationship between IL-36 and Breg subsets. Therefore, these results can provide better understanding for the overexpression and proinflammatory role of IL-36α in patients with SLE. 

## Supplementary Materials

Supplementary materials can be accessed at: http://www.mdpi.com/1420-3049/20/10/19588/s1.
