# Supplementary material for: Elevated Expression and Pro-Inflammatory Activity of IL-36 in Patients with Systemic Lupus Erythematosus
Source: Molecules. 2015 Oct 27;20(10):19588–604. doi: 10.3390/molecules201019588 (PMC6332178; doi:10.3390/molecules201019588)
Supplement: Supplementary file 1 [file molecules-20-19588-s001.pdf]

Supplementary Data

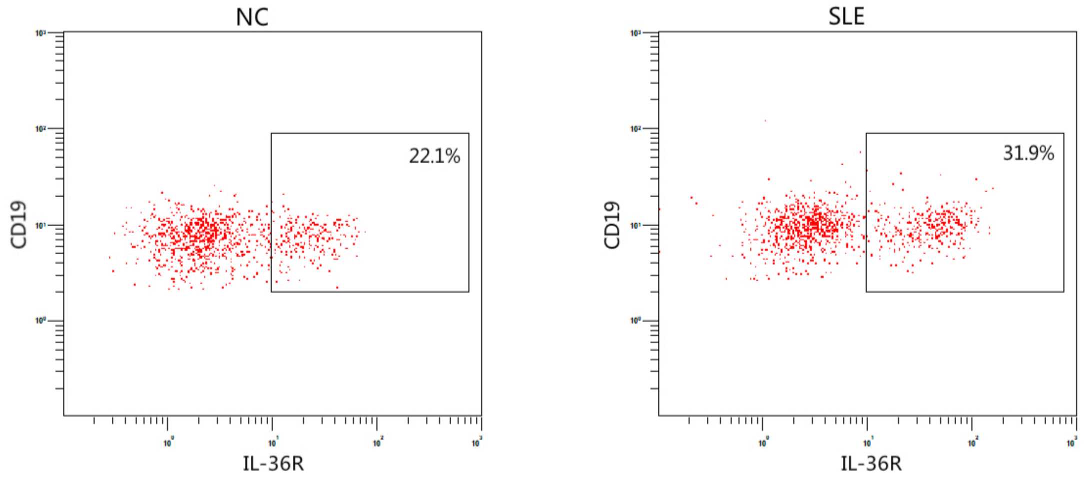

Figure S1. Representative flow cytometry dot plots of CD19$^+$IL-36R$^+$ lymphocytes gated from CD19$^+$ B cells in NC and SLE patients.

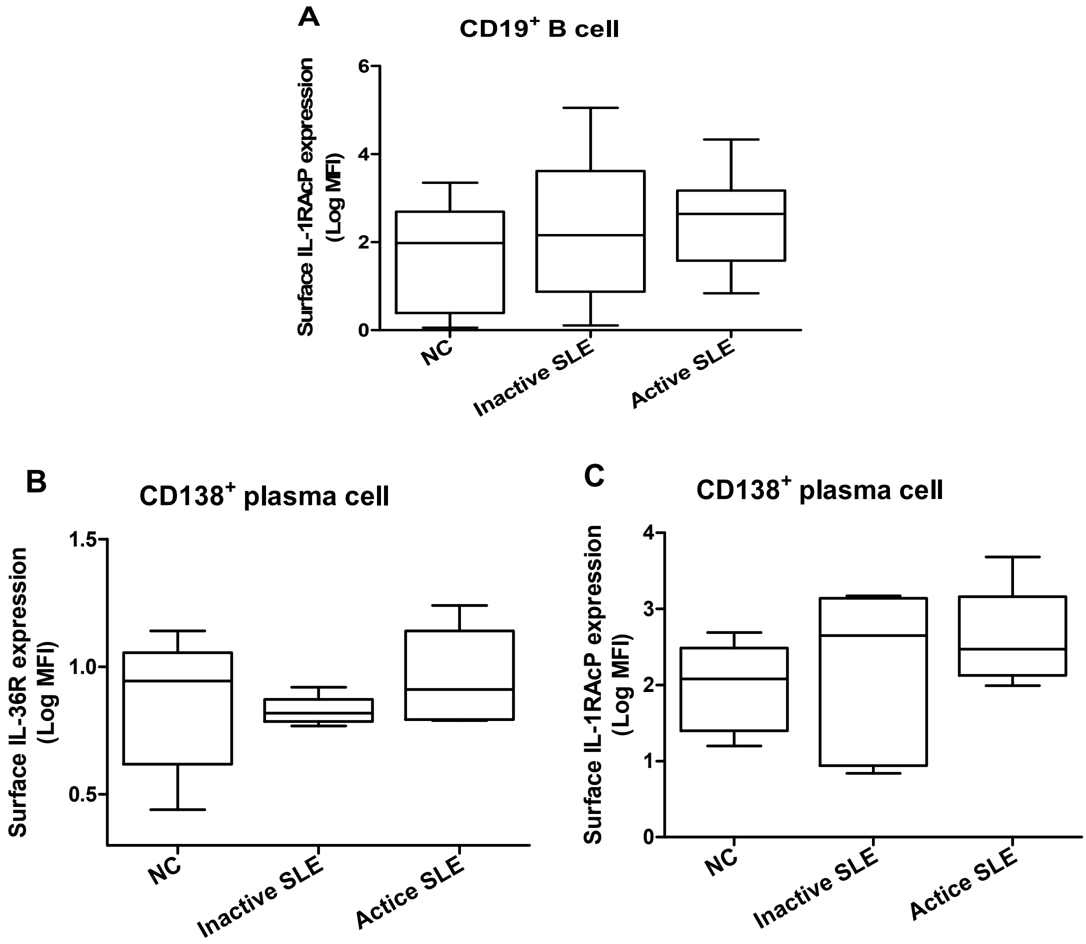

Figure S2. Comparison of expression level of IL-36 receptors on immune cells. (**A**) The expression levels of IL-1RAcp on CD19+ B cells from inactive ($n = 22$), active ($n = 21$) SLE patients and NC ($n = 16$); and (**B**,**C**) IL-36R and IL-1RAcp on CD138+ plasma cells from inactive ($n = 5$), active ($n = 5$) SLE patients and NC ($n = 5$) were detected using flow cytometry. Results are presented as box and whisker plots with median (interquartile range) of mean fluorescence intensity (MFI) subtracting corresponding isotypic controls.

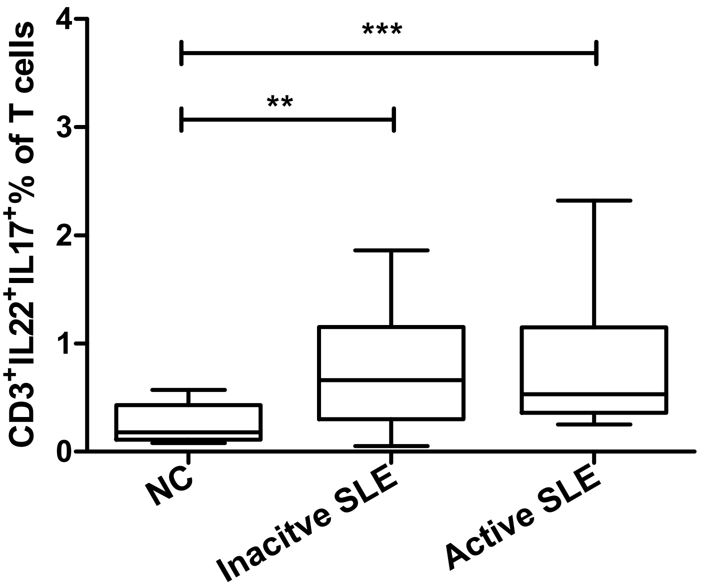

Figure S3. Comparison of circulating CD3$^+$IL-22$^+$IL-17$^+$ T lymphocytes frequency between SLE patients and NC.PBMC (2×10^6 cells/mL) were pre-incubated with 50 ng/mL phorbol myristate acetate (PMA) and 1 μg/mL ionomycin overnight, followed by treatment with 10 μg/mL Brefeldin A (BFA) for 4 h. Cells were fixed and Fc receptors were blocked with normal mouse serum. The proportion of circulating CD3$^+$IL-22$^+$IL-17$^+$ T lymphocytes from inactive (n = 22), active (n = 21) SLE patients and NC (n = 16) were determined by flow cytometry. Results are presented as box and Whisker plots with median (interquartile range). Statistical significances are indicated by ** $p < 0.01$ and *** $p < 0.001$ when compared with NC (Mann-Whitney U test).

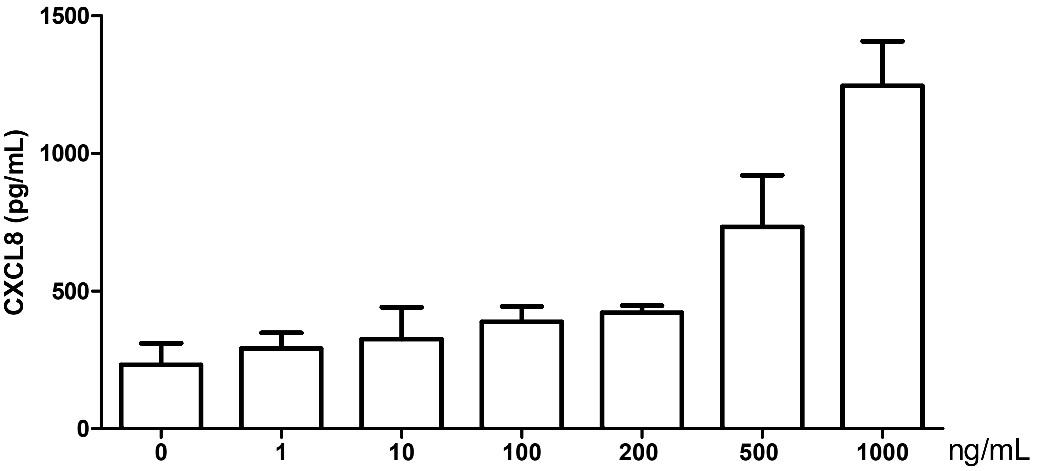

Figure S4. *Ex vivo* production of CXCL8 from PBMC stimulated with IL-36α at different concentrations for 24 h. n = 3.
